# Understanding the biopsychosocial knee osteoarthritis pain experience: an ecological momentary assessment

**DOI:** 10.1097/PR9.0000000000001172

**Published:** 2024-07-12

**Authors:** Mark Overton, Nicola Swain, Carrie Falling, David Gwynne-Jones, Roger Fillingim, Ramakrishnan Mani

**Affiliations:** a Centre for Health, Activity and Rehabilitation Research, School of Physiotherapy, University of Otago, Dunedin, New Zealand; b Department of Surgical Sciences, Otago School of Medicine, University of Otago, Dunedin, New Zealand; c Pain Research and Intervention Center of Excellence (PRICE), Department of Community Dentistry and Behavioural Science, University of Florida, Gainesville, FL, USA

**Keywords:** Knee osteoarthritis, Biopsychosocial, Pain, Smartphone, Ecological momentary assessment

## Abstract

**Introduction:**

Psychological, social, and lifestyle factors contribute to the knee osteoarthritis (OA) pain experience. These factors could be measured more accurately using smartphone ecological momentary assessment (EMA).

**Objectives:**

The objective of this study was to characterise the pain experiences of those with knee OA by a smartphone EMA survey and explain how momentary psychological and social states influence knee OA pain experiences.

**Methods:**

A smartphone EMA survey was designed and piloted. Eligible participants completed smartphone EMA assessing the knee OA pain experience 3 times daily for 2 weeks. Descriptive statistics were used to characterise factors involved in knee OA pain followed by the development of mixed-effects location scale models to explore heterogeneity and relationships between symptoms involved in the knee OA pain experience.

**Results:**

Eighty-six community-dwelling volunteers with knee OA were recruited. Pain, psychosocial, and lifestyle factors involved in knee OA pain experience were heterogeneous and variable. Those with greater variability in pain, fatigue, negative affect, and stress had worse levels of these symptoms overall. In addition, fatigue, negative affect, stress, anxiety, loneliness, and joint stiffness demonstrated within-person relationships with knee OA pain outcomes.

**Conclusions:**

Knee OA pain is a heterogeneous biopsychosocial condition. Momentary experiences of psychological, social, fatigue, and joint stiffness explain individual and between-individual differences in momentary knee OA pain experiences. Addressing these momentary factors could improve pain and functional outcomes in those with knee OA. Validation studies, including individuals with more severe knee OA presentations, are required to support findings and guide clinical interventions to improve outcomes for those with knee OA.

## Abstract 2

Supplemental Digital Content is Available in the Text.

Joint pain, psychological, social, and lifestyle factors are variable for individuals with knee osteoarthritis (OA). These momentary psychosocial and lifestyle factors contribute to the knee OA pain experience.

## 1. Introduction

Knee osteoarthritis (OA) is a prevalent musculoskeletal condition that affects approximately 16% of the adult population.^17^ Knee OA pain and disability are complex with biological, psychological, social, and lifestyle factors playing a role in knee OA lived experiences.^27,40,48^ An assessment method that may better and more accurately capture the range of symptoms and experiences involved in the knee OA pain experience is the ecological momentary assessment (EMA).^76,92,97^ A defining characteristic of this research method is that measures are repeated multiple times daily to reveal patterns of transient, fluctuating symptoms such as pain, fatigue, and mood in real-time, real-life contexts.^64,65,76,92,97^ Ecological momentary assessment methods continue to evolve, relying less on paper diaries and more on technology, including smartphones and wearable devices. These technological advancements have further assisted with the robustness of the data collected.^64,101^ Strengths of EMA include its ability to reduce recall bias and measurement errors while also offering greater ecological validity with measurement occurring in the environment and context of patients' lives.^65,92,97,98^ Furthermore, repeated measurements collected during EMA allow for within-person relationships to be explored in “real-life” contexts.^64,65^

Knee OA pain outcomes traditionally include collecting recalled average pain ratings. However, solely considering recalled averages has the potential to overlook the dynamic, fluctuating nature of symptoms in individuals with painful knee OA.^1,2,6,20,31,35,47,53,60,64,77,81,82,87,88^ Symptom variability is a normal part of the pain experience. Because EMA involves repeatedly measuring the dynamic pain experience over time, within-person variability can be explored.^20,34,35,65,82^ Exploring within-person variability in knee OA symptoms may reflect a different, yet clinically important aspect of the knee OA pain experience.^34^ Especially considering that variability in pain intensity has been linked with disability, work, sleep, psychological, and quality of life outcomes.^1,2,4,6,41,47,53,60,77,81,82,88^

Studies are yet to widely use EMA methods to explore the pain experiences of those with knee OA.^24,28,54,81,94,107^ In the few EMA studies that have been performed on this population, these focus on fatigue and self-efficacy and their impact on functioning.^24,69,71,72,94,103,107^ In addition, daily pain and functioning have also been explored in mixed OA populations, for short durations and using nonrandom EMA methods.^2,28,81^ To the best of our knowledge, EMA studies are yet to capture the biopsychosocial knee OA pain experience and explore within-person relationships using smartphone technology. A better understanding of momentary psychosocial and lifestyle factors and how these influence knee OA pain experiences would provide a more in-depth understanding of the complex, dynamic factors involved in knee OA pain and identify potential targets for treatment.

Therefore, this study aims to characterise the pain experiences of those with knee OA by a smartphone EMA survey and explain how momentary psychological and social states influence knee OA pain experiences. This exploratory investigation was carried out in 2 stages. Stage 1: Develop and pilot a smartphone EMA survey to explore the knee OA pain experience. Stage 2: Using the smartphone EMA survey, characterise the pain experiences of those with knee OA and explain how psychological and social states influence knee OA pain experiences.

### 1.1. Objectives

(1) To develop and pilot a prototype of a smartphone EMA survey to assess usability, clarity, and time to complete the survey in a sample of individuals with knee OA.
(2) To characterise the biopsychosocial knee OA pain experience using smartphone EMA.
(3) To determine within-person variability (scale) and its relationship with within-person mean (location) in knee OA symptoms.
(4) To examine whether participants' momentary psychological and social states demonstrate relationships with momentary pain intensity, interference, and bothersomeness.

## 2. Methods

### 2.1. Study design

This article presents findings from a 2-week smartphone EMA study which was developed in consultation with the Checklist for Reporting Ecological Momentary Assessment Studies (CREMAS) and EMA literature.^14,55,91,99,100^ Ethical approval was obtained from the Central Health and Disability Ethics Committee of New Zealand (21/CEN/89).

### 2.2. Participants

Participants were eligible for inclusion if aged 45 to 85 years with a diagnosis of knee OA and had experienced knee pain on most days for at least 3 months. Participants fulfilling NICE guidelines for a clinical diagnosis of knee OA were also included.^74^

Participants were excluded if they reported being a non-English speaker, unable to use a smartphone, had other rheumatological and autoimmune conditions, had uncontrolled hypertension, skin conditions, lower limb sensory loss, had undergone or were scheduled for knee arthroplasty, had a separate leg injury, had a neurological condition, impaired cognition, or psychiatric illness.

Participants were recruited from hospital outpatient settings and the community. Participants involved in this study were provided with a $100 supermarket voucher to recognise any costs involved with participation.

### 2.3. Baseline assessment

Participant characteristics, including demographic information (age, sex, ethnicity, knee OA duration, educational level, residential address, and work status), were collected. The Montreal Cognitive Assessment (MoCA) was administered to participants to detect mild cognitive impairment.^73^ Participants scoring <16 were excluded from this study.^67^

#### 2.3.1. Stage 1: development and piloting of survey

A narrative review of the literature identified pain-related measures and psychosocial, behavioural, and lifestyle constructs related to the knee OA pain experience.^10–13,19,23,28–30,32,36,37,44,45,50,52,56,62-64,84,103–105^ Validated single-item measures or single-item measures used in previous published EMA studies were used to reduce participant burden.^10–13,19,23,28–30,32,36,37,44,45,50,52,56,62–64,84,103–105^ Primary variables of interest included pain intensity and pain interference. Additional variables included pain bothersomeness, physical activity, sedentary time, flare-up status, positive and negative affect, stiffness, sleep quality, fatigue, stress, anxiety, social contact, and loneliness. A preliminary EMA survey and protocol was developed (Table 1), which included EMA for 2 weeks, whereby participants received a survey 3 times daily.

**Table 1:** U-KOPE ecological momentary assessment survey items and schedule.

EMA question	Morning	Day	Evening	Response
What is your level of pain right now?^22,88^	✓	✓	✓	11-point NRS (0 = No pain, 10 = Worst pain imaginable)
How much is your pain interfering with what you are doing right now?^22^	✓	✓	✓	11-point NRS (0 = No interference, 10 = Totally interfering)
How bothersome is your knee pain currently?^32,87^	✓	✓	✓	5-item ordinal scale (Not at all, Slightly, Moderately, Very Much, Extremely)
How many hours did you spend sitting yesterday (eg, sitting at work, watching TV, driving, seated leisure)?^53,74^	✓			Numeric
Have you done 30 minutes or more of physical activity today, which was enough to raise your breathing rate (ie, sport, exercise, brisk walking, cycling)?^48^			✓	Yes/No
Have you experienced an osteoarthritis flare-up today (“… different from usual state… worsening of pain, swelling, stiffness which impacts on sleep, activity, functioning and psychological aspects…”)?^46^			✓	Yes/No
How severe is your knee stiffness currently?^16,17^	✓	✓	✓	5-item ordinal scale (None, Mild, Moderate, Severe, Extreme)
What best describes how well you slept last night?^53,140^	✓			5-item ordinal scale (Very Bad, Bad, Fair, Good, Very Good)
What number best describes how fatigued or tired you are right now?^140^	✓	✓	✓	11-point NRS (0 = Not fatigued, 10 = Fatigued as bad as I can imagine)
Please rate your current level of happiness^15,52,53,72^	✓	✓	✓	11-point NRS (0 = Not at all, 10 = Extremely)
Please rate your current level of frustration^15,52,53,72^	✓	✓	✓	11-point NRS (0 = Not at all, 10 = Extremely)
Are you currently experiencing feelings of panic, worry, or anxiety?^53,72,113^	✓	✓	✓	5-item ordinal scale (Not at all, Slightly, Moderately, Very Much, Extremely)
How much stress do you feel right now?^44,45,79^	✓	✓	✓	11-point NRS (0 = No stress, 10 = Extreme stress)
Who are you currently with?^15,37,45,86,142^	✓	✓	✓	Multiple choice (Alone or with strangers only, Spouse/partner, Children, Other family, Colleagues, Clients/customers, Friends, Other people you know)
Please rate your current level of loneliness^63,64,144^	✓	✓	✓	5-item ordinal scale (Not at all, Slightly, Moderately, Very Much, Extremely)

EMA, ecological momentary assessment; NRS, numeric rating scale; U-KOPE, understanding knee osteoarthritis pain experiences.

The objectives of the EMA piloting process included improving survey usability, question clarity, and limiting participant burden. Five volunteers with knee OA were screened for eligibility and, following training, were invited to complete the first version of the smartphone EMA survey and provide feedback by a questionnaire and through the “think-aloud” method, where participants immediately verbalised their thoughts.^43^ The primary investigator recorded “think-aloud” data using an audio recorder. Written feedback was also sought on the survey duration, question clarity, comprehensiveness, or any other barriers. Clarity was rated on an 11-point scale where 0 was “Not at all clear” and 10 was “Extremely clear.” Participants were also able to provide free-text comments.

Audio from the “think-aloud” process was transcribed verbatim. Transcripts were reviewed to identify common themes, and written feedback was considered to inform the final survey design. The mean numeric clarity rating was calculated with ratings below 7/10, resulting in the question being amended.

#### 2.3.2. Stage 2: administering the smartphone ecological momentary assessment survey

**Figure 1.**
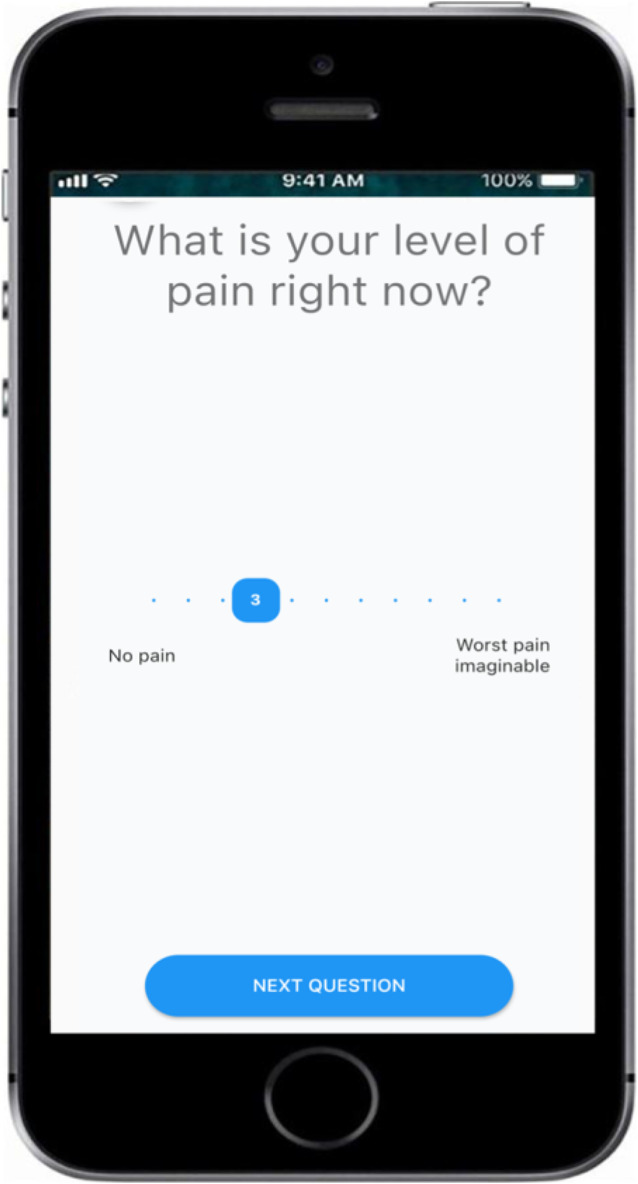
Screenshot of the smartphone EMA survey using the m-Path application. EMA, ecological momentary assessment.

Participants underwent 10 to 15 minutes of EMA training to aid in familiarising themselves with the smartphone, the EMA application, and survey questions and to ensure that survey notifications were being received. Participants were either provided with a smartphone or could choose to use their own device and download the freely available m-Path application (Fig. 1). The researcher-provided smartphone was a Nokia 2.3, Nokia Corporation which used an Android 12 operating system (Snow Cone). In participants choosing to use their own smartphone, a range of devices were used. Following training, participants were provided with an instructional handout and signed a statement of commitment.

Eligible volunteers participated in 14 consecutive days of smartphone EMA monitoring (one wave; 10 weekdays and 4 weekend days).^64^ Participants were required to complete the smartphone survey 3 times daily.^49,66^ Ecological momentary assessment prompting occurred in a random-stratified manner, with participants being notified randomly within 3 prespecified time blocks throughout their day. This ensured that symptoms after waking, during the day, and in the evening were collected to get a representative dataset. The random-stratified blocks were scheduled as follows:

(1) Morning: A 2-hour block was placed immediately following the usual wake time.
(2) Day: A 5-hour block was placed from 11 am (or 2 hours following usual wake time).^61^
(3) Evening: A 2-hour block was placed immediately before the usual bedtime.

Therefore, each participant was sent 42 surveys to complete during the study. Latency was <60 minutes, with responses >60 minutes being considered missing. Reminder prompting after 30 minutes and “snooze” features for up to 60 minutes were also incorporated to improve compliance.^55^ Question ordering differed between morning, afternoon, and evening surveys with prior responses not being viewable.

### 2.4. Data management

The full data management and analysis plan can be found in Appendix A, http://links.lww.com/PR9/A236. Missing data trends for EMA data were analysed to determine potential patterns of missingness.^7^

### 2.5. Statistical analysis plan

A series of multilevel mixed-effects location scale (MELS) models were performed using Mixed models With Intensive Longitudinal Data (MixWILD), Version 1 allowing for the assessment of within-person location (mean) and scale (variability) effects.^22,33^ Empty MELS models (models without predictors) were completed to calculate the random log-transformed scale standard deviation and location–scale relationship. Mixed-effects location scale models were then completed, exploring the within- and between-person effects of momentary fatigue, stiffness, loneliness, negative affect, anxiety and stress on pain intensity, interference, and bothersomeness outcomes. The level of error considered acceptable for statistical significance was set at *P* ≤ 0.05.

## 3. Results

### 3.1. Objective 1: ecological momentary assessment piloting outcomes

A prototype of the smartphone EMA survey was piloted on 5 participants with knee OA. Four of the pilot participants were men. These participants had a mean age of 58.2 ± 11 years and reported experiencing knee OA for a mean duration of 8 ± 7.4 years. Overall, the smartphone EMA survey was endorsed with no significant issues reported. Clarity ratings for the first version of the smartphone EMA survey are presented in Table 2.

**Table 2:** Clarity ratings for preliminary smartphone survey items.

Smartphone EMA construct measured	Clarity rating*
Pain intensity	9.8 ± 0.4
Pain interference	9.8 ± 0.4
Pain bothersomeness	5.6 ± 3.3
Stiffness	9.4 ± 1.3
Fatigue	10 ± 0
Sleep quality	9.8 ± 0.4
Positive affect	10 ± 0
Negative affect	9.8 ± 0.4
Anxiety	10 ± 0
Stress	10 ± 0
Social contact	9.8 ± 0.4
Loneliness	10 ± 0
Flare-up	9 ± 1.4
Physical activity	9.8 ± 0.4
Sedentary time	9.4 ± 1.3

* Data are presented as mean ± standard deviation.

EMA, ecological momentary assessment.

Participants estimated that the average time to complete the survey was 4.9 ± 3.1 minutes. All participants deemed smartphone EMA and the questions as being acceptable and relevant to their knee OA experiences. One recommendation included asking about specific activities that pain had interfered with. This was, therefore, included as a checkbox item in the final EMA survey. Three of the 5 participants (60%) reported that the original pain bothersomeness question was confusing and difficult to understand. Therefore, “Before prompt” was removed and replaced with “Currently.” All included pilot participants reported that they would prefer to use their own smartphones.

### 3.2. Objective 2: characterising knee osteoarthritis using smartphone ecological momentary assessment

#### 3.2.1. Participant characteristics

**Figure 2.**
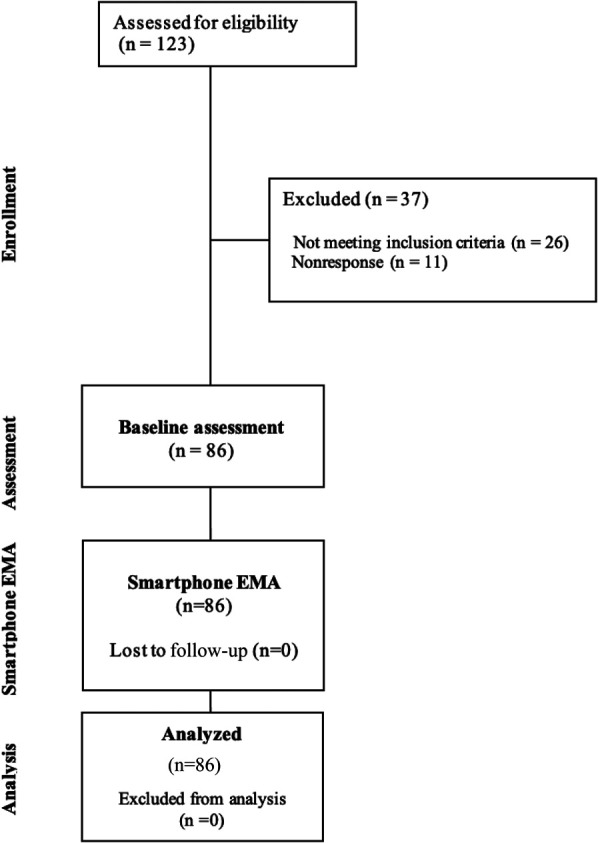
Participant flow diagram.

A final sample size of 86 participants was included, with no loss to follow-up. The participant flow diagram is presented in Figure 2.

Characteristics of study participants are presented in Table 3.

**Table 3:** Characteristics of included participants.

Characteristic	Value*
Age (y)	67.3 ± 9.1
Sex	Female: 55 [64] Male: 31 [36]
Ethnicity	NZ European: 78 [90.6] New Zealand Māori: 4 [4.7] Indian: 2 [2.3] English European: 1 [1.2] Egyptian: 1 [1.2]
BMI (kg/m^2^)	32 ± 6.8
Handedness	Right: 80 [93] Left: 6 [7]
Knee OA duration (y)	9.2 ± 9.1
Bilateral OA	Yes: 48 [55.8] No: 38 [44.2]
Worst knee	Right: 46 [53.5] Left: 40 [46.5]
Highest level of education	No formal qualification: 12 [14] Year 10: 1 [1.2] Year 13: 12 [14] Trade/apprenticeship: 7 [8.1] Certificate/diploma: 19 [22.1] University degree: 19 [22.1] Postgraduate degree: 16 [18.6]
Work status	Full-time employed: 21 [24.4] Part-time employed: 9 [10.5] Self-employed: 7 [8.1] Homemaker: 1 [1.2] Retired: 47 [54.7] Unable to work: 1 [1.2]

* Data are presented as mean ± standard deviation or number [%].

BMI, body mass index; kg, kilograms; m, metres; OA osteoarthritis.

#### 3.2.2. Ecological momentary assessment participation and compliance

Average compliance across the two-week monitoring period was 90.7% ± 8.8. Eighty participants (93%) used their own phones. All participants completed the study.

Nonparametric correlations (Kendall Tau and Spearman) were completed to explore whether compliance was related to demographic variables. Only gender demonstrated a fair relationship with EMA compliance: with women responding to more of the smartphone EMA surveys (r = 0.3, *P* = 0.02). Nonresponse was deemed to be MAR with no evidence of relationships between data missingness and variables including pain intensity (*P* = 0.4), pain interference (*P* = 1), fatigue (*P* = 0.4), negative affect (*P* = 0.5), and the number of flare-up days (*P* = 0.9). There was no evidence of measurement reactivity with no statistically significant change in pain intensity ratings between weeks of EMA monitoring (*P* = 0.5).

#### 3.2.3. Aggregated ecological momentary assessment measures

The repeated measures from the smartphone EMA allowed for the aggregation of pain intensity ratings.^87^ Aggregation was also performed for other constructs collected. Aggregated data are presented in Table 4.

**Table 4:** U-KOPE aggregated ecological momentary assessment data.

Aggregated measure	Mean ± SD
Pain intensity	
Current	2.7 ± 1.9
Maximum	5.6 ± 2.3
Minimum	0.9 ± 1.2
Variability (SD)	1.2 ± 0.5
Time high (%)	3.3 ± 11.2
Time low (%)	65.2 ± 28.1
Pain interference	
Current	2 ± 1.6
Maximum	5.2 ± 2.7
Minimum	0.3 ± 0.6
Variability (SD)	1.3 ± 0.7
Time high (%)	2.1 ± 6.5
Time low (%)	73.2 ± 22.1
Pain bothersomeness (total number [%])	
Not at all	725 [22.1]
Slightly	1533 [46.8]
Moderately	836 [25.5]
Very Much	167 [5.1]
Extremely	16 [0.5]
Knee OA flares (total number [%])	
Total flare days	265 [24.4]
Average flare days per person	3.1 ± 3
Stiffness (total number [%])	
None	682 [20.8]
Mild	1515 [46.2]
Moderate	951 [29]
Severe	120 [3.7]
Extreme	9 [0.3]
Fatigue	
Current	3.4 ± 1.9
Maximum	6.8 ± 2.1
Minimum	0.8 ± 1.1
Variability (SD)	1.7 ± 0.7
Time high (%)	6.1 ± 10.9
Time low (%)	52.7 ± 29.7
Sleep quality (total number [%])	
Very bad	25 [2.2]
Bad	114 [10.2]
Fair	406 [36.5]
Good	435 [39.1]
Very good	133 [12]
Positive affect	
Current	7.5 ± 1.3
Maximum	9 ± 1
Minimum	4.5 ± 2.5
Variability (SD)	1.1 ± 0.6
Time high (%)	53.9 ± 33.3
Time low (%)	3.2 ± 6.8
Negative affect	
Current	2.1 ± 1.7
Maximum	5.9 ± 2.8
Minimum	0.4 ± 0.7
Variability	1.4 ± 0.8
Time high (%)	3.4 ± 8.9
Time low (%)	72.2 ± 25.2
Anxiety (total number [%])	
Not at all	2424 [74]
Slightly	725 [22.1]
Moderately	114 [3.5]
Very Much	14 [0.4]
Extremely	0
Stress	
Current	1.8 ± 1.6
Maximum	4.8 ± 2.8
Minimum	0.3 ± 0.7
Variability (SD)	1.1 ± 0.7
Time high (%)	1.8 ± 6.9
Time low (%)	77.1 ± 22.8
Loneliness (total number [%])	
Not at all	3086 [94.2]
Slightly	153 [4.7]
Moderately	30 [0.9]
Very Much	8 [0.2]
Extremely	0
Physical activity	
30 min achieved	639 [58.8]
Average days PA achieved per person	7.4 ± 4.1
Sedentary time (h)	
Daily sedentary time	6.6 ± 2.3
Maximum	8.7 ± 2.9
Minimum	4.2 ± 1.8
Variability (SD)	1.4 ± 0.6

Variability is the average of each participant's standard deviation. Time in high (%) is the percentage of ratings ≥7.5/10. Time in low (%) is the percentage of ratings ≤3.5/10.

PA, physical activity; SD, standard deviation; U-KOPE, understanding knee osteoarthritis pain experiences.

### 3.3. Objective 3: knee osteoarthritis symptom variability

Using MixWILD, variability in scale and associations between within-person means and standard deviations (location–scale relationships) were explored. Findings are presented in Table 5.

**Table 5:** Variability in scale and location–scale relationships for ecological momentary assessment variables.

EMA variable	Random scale SD*	Random location† effect on scale
Pain intensity	0.7***	0.6***
Pain interference	1.4***	−0.1
Pain bothersomeness	0.3***	0.1
Stiffness	0.4***	0
Fatigue	0.7***	0.3***
Negative affect	0.9***	0.6***
Anxiety	0.4***	0
Stress	1***	0.9***
Loneliness	0.5**	0.3
Positive affect	1.1***	−0.4**
Sleep quality	0.3***	−0.2**
Sedentary time	0.6***	0.5***

***P < 0.001; **P < 0.01.

* Random scale SD reflects variability in scale represented as log-transformed standard deviation.

† Random location reflects within-in participant mean.

EMA, ecological momentary assessment; SD, standard deviation.

Participants significantly differed from one another in terms of their symptom variability for all measures collected by smartphone EMA. Location–scale relationships were demonstrated for pain intensity, fatigue, negative affect, stress, and sedentary time meaning that those reporting greater average levels, also demonstrated greater variability in these variables. Alternatively, inverse location–scale relationships were demonstrated for positive affect and sleep quality, meaning that better average positive affect and sleep quality were associated with less variability in these variables. Nonsignificant location–scale relationships were found for pain interference, bothersomeness, and anxiety.

### 3.4. Objective 4: influence of momentary psychosocial and lifestyle factors on knee osteoarthritis pain experiences

The relationship between momentary psychosocial and lifestyle factors and the pain experiences of those with knee OA was explored. These are presented in Table 6.

**Table 6:** Effect of ecological momentary assessment variables on knee osteoarthritis pain outcomes.

Variable	Effect	ß—pain intensity	ß—pain interference	ß—pain bothersomeness
Fatigue	Between Within	0.6*** 0.2***	0.5*** 0.2***	0.9*** 0.3***
Negative affect	Between Within	0.5*** 0.3***	0.5*** 0.4***	0.7*** 0.5***
Stiffness	Between Within	1.5*** 1.1***	1.5***,‡ 1.1***,‡	3.6*** 3***
Anxiety	Between Within	0.3 0.4***	0.3† 0.2***,†	1.2* 0.5***
Stress	Between Within	0.5*** 0.3***	0.5*** 0.4***	0.7*** 0.4***
Loneliness	Between Within	0.4**,† 0†	2.2***,‡ 0.4**,‡	2.5* 0.4*

***P < 0.001; **P < 0.01; *P < 0.05.

Between-person estimate represents a participant's average level relative to the group mean.

Within-person estimate represents participants' momentary ratings relative to their own mean.

† Standardised.

‡ Scale parameters.

#### 3.4.1. Pain intensity

Within-person relationships were demonstrated between pain intensity and fatigue, negative affect, knee stiffness, stress, and anxiety. Between-person relationships were demonstrated between pain intensity and fatigue, negative affect, knee stiffness, and stress.

#### 3.4.2. Pain interference

Within-person relationships were demonstrated between pain interference and fatigue, negative affect, stress, anxiety, and loneliness. Between-person relationships were demonstrated between pain interference and fatigue, negative affect, stress, and loneliness.

#### 3.4.3. Pain bothersomeness

Both within- and between-person relationships were demonstrated between pain bothersomeness and fatigue, negative affect, stress, anxiety, and loneliness.

## 4. Discussion

The aim of this study was to characterise the pain experiences of those with knee OA by smartphone EMA and explain how participants' momentary psychological and social states and other experiences, such as fatigue and joint stiffness, influence pain experiences. This study showed that the knee OA pain experience was variable and unique to the individual. In addition, the knee OA pain experience was shown to be influenced by individual psychosocial and lifestyle factors.

Symptom variability is a normal part of the pain experience, demonstrated in several populations.^1,2,6,47,53,60,77,81,88^ This study adds to previous findings, demonstrating symptom heterogeneity in those with knee OA using robust smartphone EMA methods.^2,41,80^ Incidentally, greater variability in many knee OA symptoms (ie, pain intensity) was related to greater average symptom levels overall highlighted by location–scale relationships. Studies across multiple patient populations have shown that pain variably is related to poorer function, quality of life, sleep, work absence, and psychological and health resource use outcomes.^4,41,88^ This suggests that targeting pain variability may result in improvements across multiple patient outcomes.^4,41,88^ Furthermore, as pain variability is typically unpredictable and can cause a decrease in locus of control, patients may cope better if they experienced less pain variability.^88^ Management strategies which address pain aggravations such as proactive supported self-management strategies, analgesia regimes, and psychological coping interventions may be beneficial.^2,41^ However, further research exploring whether the impact of pain variability can be improved in those with knee OA is required.^2,88^

Most participants reported mild pain across the two-week monitoring period. Minimal time in high pain (≥7.5/10 on the NPRS) was reported, with the participants reporting low pain (≤3.5/10 on the NPRS) most of the time. Although time in high pain was low in this study, this may have important clinical consequences. A recent meta-analysis demonstrated relationships between time in high pain and clinical outcomes including worse functioning which could negatively impact quality of life.^77,87^ Furthermore, time in high pain may reflect the presence of knee OA flare-ups or underlying pain mechanisms such as sensitization.^87^ Identifying those with more time in high pain may assist with prioritizing those in greatest need of intervention, subgrouping the knee OA population, and be an important target for treatment.^87^ More research is required to explore the implications of time in high pain and better address this to improve clinical outcomes.

In this study, participants rated whether they experienced a flare-up day. On average, participants reported of having a flare-up day on almost 25% of the two-week monitoring period. This suggests that flare-up occurrence reported by previous studies (2.4 annually) may underestimate the incidence of OA flare-ups.^8^ In addition, participants may have rated even minor symptom interference based on the provided definition of OA flare-ups. This may reflect heterogeneity in OA flare-up presentations with different severities and durations of flare-ups presenting in the OA population. Flare-ups play a significant role in the knee OA pain experience, contributing to functional, sleep, and psychological consequences.^30^ Reducing the magnitude and number of flare-ups could significantly reduce disability and improve quality of life. Consequently, future research should investigate treatments that target OA flares, including triggers and outcomes.

Fatigue is a complex, multifactorial symptom experienced by those with knee OA which negatively impacts functioning and quality of life.^25,26,70–72^ A recent systematic review reported that factors contributing to fatigue in this population include lower levels of functioning, comorbidities, pain, sleep quality, and depression.^85^ Fatigue was highlighted by this study as a heterogeneous symptom that also contributes to the knee OA pain experience. Factors contributing to fatigue in the knee OA pain experience include systemic inflammation, prolonged sympathetic nervous system activity, and sensitization making fatigue, pain, and sleep quality interrelated symptoms.^5,21,51,57,78,79,83,86,95,102,103^ With these many complex factors contributing to the knee OA presentation, a vicious cycle of worsening pain, fatigue, and disability could develop endorsing the need for intervention.^25,26,85,103^

Psychological factors play an important role in predicting pain and functional outcomes in those with knee OA.^16,38,39,90,96^ In this study, most participants reported low overall levels of negative affect, anxiety, and stress; however, these symptoms were shown to be heterogeneous across the sample. Those who reported worse mood and stress were shown to have greater symptom variability. By contrast, those reporting greater positive mood presented with less variability in positive mood. When compared with pain outcomes, participants who reported worse-than-usual mood, anxiety, and stress also reported higher pain intensity, interference, and bothersomeness. A recent review confirms the high prevalence of anxiety and depression in those with knee OA, with psychological factors being associated with greater levels of pain and disability.^42,58,89^ In addition, greater pain and disability have been shown to prospectively predict the incidence of future depression.^108^ These findings highlight that psychological factors may be strongly related to an individual's knee OA pain experience, whereby knee OA pain, disability, depression, distress, and anxiety could influence each other, contributing to a vicious cycle.^106^

Chronic pain, disability, and depression are risk factors for social isolation and loneliness.^75,93,96^ Therefore, those with knee OA may be at greater risk. In this study, loneliness was shown to be variable and demonstrated relationships with worse pain intensity, interference, and bothersomeness. Knee OA pain may limit individuals from socialising while reduced social participation may result in functional decline and increased knee OA symptoms.^46^ Consequently, interventions that aim to improve social connection and perceived loneliness may be worthwhile to improve pain, functioning, and psychological status in those with knee OA.^75,93,96^

The findings from this study suggest that targeting symptom stability, reducing time in high pain, and improving lifestyle and psychosocial status may improve knee OA symptoms, functioning, and quality of life. Interventions such as prophylactic analgesic medication regimes and the implementation of proactive self-management strategies, psychological coping strategy implementation, physical activity prescription, sleep hygiene interventions, and meditation or relaxation could be effective in improving the knee OA pain experience.^43–45^

The strengths of this study included study design adherence to the CREMAS to limit potential sources of bias^55^ and high compliance of study participants. In addition, pain, psychosocial, and lifestyle factors were captured via a momentary (or close to momentary) single-item questionnaire. This allowed these constructs to be collected in a manner that reduced the burden and requirements for recall. The survey items were piloted on a small group of study participants with written and “think-aloud” feedback collected to inform the final EMA survey design. Design features to reduce participant burden and improve the quality of the data were also incorporated (ie, training, individualised notification schedule, reminder or snooze features, use of own devices) to enhance the EMA experience. Other novel areas explored in this investigation include the role of loneliness, an important social construct on momentary pain experiences, suggesting a potential role of social referral as part of the holistic management of knee OA.^15^ Finally, a multilevel modelling approach using the recently developed MELS model was used to analyse the repeated, nested data and allow for the exploration of the novel within- and between-person relationships involved in the biopsychosocial knee OA pain experience.^22,53^

This study solely included participants from the community who mainly presented with a mild knee OA presentation on average. Despite attempts, no participants were recruited from the hospital's orthopaedic outpatient department. Reports suggest that individuals with more severe symptoms may present with more variable symptoms, including the presence of additional psychosocial factors.^3,18^ In this study, more mild presentations likely meant that there was less potential distribution of variables. This inevitably meant that participants with a milder presentation overall would have lower momentary reports of symptoms, resulting in less variability. Therefore, further research on knee OA populations with moderate and severe knee OA pain experiences which controls for overall pain levels is needed to fully conclude that variability is the driving factor. Study participants presented with a large degree of variability in knee OA duration. Including knee OA duration in future MELS models may suggest the possible impact that knee OA duration has on pain experiences. The power analysis completed was not specific to the EMA data and MELS modelling. However, within-person analyses are often reported to improve power.^59,68^ Therefore, the included sample was larger than many EMA studies exploring populations with pain.^64,76^ With this being an exploratory study, larger and longer validation studies are required to support findings and further explore the statistical and clinical significance of demonstrated relationships. Testing hypotheses informed by this study using lagged effect models is also required.

Ecological momentary assessment surveys were only administered 3 times daily. Therefore, these may have potentially missed times of high or low symptoms. A few items included in the EMA survey did not have established psychometric properties. Although reducing the burden, using single-item questions to capture complex experiences may fail to adequately capture constructs of interest.^9^ Therefore, psychometric properties of single-item measures need to be established for use in future EMA studies.

Overall, data from this study suggest that pain, psychosocial, and lifestyle factors involved in knee OA pain experience are heterogeneous and variable in real-life contexts and circumstances. Those with greater variability in pain, fatigue, negative affect, and stress had worse levels of these symptoms overall. This highlights that knee OA is not a static, homogenous condition. Instead, knee OA differs between individuals, with symptoms fluctuating over hours and days. Furthermore, momentary psychosocial and lifestyle factors, such as low mood, stress, anxiety, and loneliness, and bodily experiences, such as fatigue and joint stiffness, were shown to demonstrate relationships with variable pain experiences at an individual level. These are important findings for clinical practice, endorsing the need to provide treatments that better manage symptom stability and address individualised psychosocial and lifestyle factors that have been shown to contribute to the knee OA pain experience.

## Disclosures

The authors have no conflict of interest to declare.

## Appendix A. Supplemental digital content

Supplemental digital content associated with this article can be found online at http://links.lww.com/PR9/A236.

## References

[1] Affleck G Tennen H Keefe FJ Lefebvre JC Kashikar-Zuck S Wright K Starr K Caldwell DS. Everyday life with osteoarthritis or rheumatoid arthritis: independent effects of disease and gender on daily pain, mood, and coping. PAIN 1999;83:601–9. 10568869 10.1016/S0304-3959(99)00167-0

[2] Allen K Coffman C Golightly Y Stechuchak K Keefe F. Daily pain variations among patients with hand, hip, and knee osteoarthritis. Osteoarthritis Cartilage 2009;17:1275–82. 19410670 10.1016/j.joca.2009.03.021

[3] Arendt-Nielsen L Nie H Laursen MB Laursen BS Madeleine P Simonsen OH Graven-Nielsen T. Sensitization in patients with painful knee osteoarthritis. PAIN 2010;149:573–81. 20418016 10.1016/j.pain.2010.04.003

[4] Bakshi N Gillespie S McClish D McCracken C Smith WR Krishnamurti L. Intraindividual pain variability and phenotypes of pain in sickle cell disease: a secondary analysis from the Pain in Sickle Cell Epidemiology Study. PAIN 2022;163:1102–13. 34538841 10.1097/j.pain.0000000000002479 PMC9100443

[5] Batushansky A Zhu S Komaravolu R South S Mehta-D’souza P Griffin T. Fundamentals of OA. An initiative of Osteoarthritis and Cartilage. Obesity and metabolic factors in OA. Osteoarthritis Cartilage 2022;30:501–15. 34537381 10.1016/j.joca.2021.06.013 PMC8926936

[6] Bellamy N Sothern R Campbell J. Rhythmic variations in pain perception in osteoarthritis of the knee. J Rheumatol 1990;17:364–72. 2332859

[7] Black AC Harel O Matthews G. Techniques for analyzing intensive longitudinal data with missing values In: Mehl MR, Conner TS, editors. Handbook of research methods for studying daily life. New York: The Guilford Press, 2012. p. 339–356.

[8] Bowden JL Kobayashi S Hunter DJ Mills K Peat G Guillemin F Parry E Thomas MJ Eyles JP. Best-practice clinical management of flares in people with osteoarthritis: a scoping review of behavioral, lifestyle and adjunctive treatments. Semin Arthritis Rheum 2021;51:749–60. 34144385 10.1016/j.semarthrit.2021.04.017

[9] Bowling A. Just one question: if one question works, why ask several? J Epidemiol Commun Health 2005;59:342–5. 10.1136/jech.2004.021204 PMC1733095 15831678

[10] Broderick JE Schwartz JE Schneider S Stone AA. Can end-of-day reports replace momentary assessment of pain and fatigue? J Pain 2009;10:274–81. 19070550 10.1016/j.jpain.2008.09.003 PMC2757263

[11] Brooks LO Rolfe MI Cheras PA Myers SP. The comprehensive osteoarthritis test: a simple index for measurement of treatment effects in clinical trials. J Rheumatol 2004;31:1180–6. 15170933

[12] Bruce B Fries J. Longitudinal comparison of the health assessment questionnaire (HAQ) and the Western Ontario and McMaster Universities osteoarthritis index (WOMAC). Arthritis Rheum 2004;51:730–7. 15478152 10.1002/art.20695

[13] Carlozzi NE Schilling S Freedman J Kalpakjian CZ Kratz AL. The reliability of end of day and ecological momentary assessments of pain and pain interference in individuals with spinal cord injury. Qual Life Res 2018;27:3003–12. 30073468 10.1007/s11136-018-1952-y PMC6196118

[14] Conner TS Mehl MR. Ambulatory assessment: methods for studying everyday life. In: Scott RA Kosslyn SM Buchmann M, editors. Emerging trends in the social and behavioral sciences: an interdisciplinary, searchable, and linkable resource. Hoboken: John Wiley & Sons, 2015. pp. 1–15.

[15] Costa A Sousa CJ Seabra PRC Virgolino A Santos O Lopes J Henriques A Nogueira P Alarcão V. Effectiveness of social prescribing programs in the primary health-care context: a systematic literature review. Sustainability 2021;13:2731.

[16] Cruz-Almeida Y King CD Goodin BR Sibille KT Glover TL Riley JL Sotolongo A Herbert MS Schmidt J Fessler BJ Redden DT Staud R Bradley LA Fillingim RB. Psychological profiles and pain characteristics of older adults with knee osteoarthritis. Arthritis Care Res (Hoboken) 2013;65:1786–94. 23861288 10.1002/acr.22070 PMC3922880

[17] Cui A Li H Wang D Zhong J Chen Y Lu H. Global, regional prevalence, incidence and risk factors of knee osteoarthritis in population-based studies. EClinicalMedicine 2020;29-30:100587. 34505846 10.1016/j.eclinm.2020.100587 PMC7704420

[18] Dell'Isola A Allan R Smith SL Marreiros SS Steultjens M. Identification of clinical phenotypes in knee osteoarthritis: a systematic review of the literature. BMC Musculoskelet Disord 2016;17:425. 27733199 10.1186/s12891-016-1286-2 PMC5062907

[19] Dunn KM Croft PR. Classification of low back pain in primary care: using “bothersomeness” to identify the most severe cases. Spine (Phila Pa 1976) 2005;30:1887–92. 16103861 10.1097/01.brs.0000173900.46863.02

[20] Dunton GF. Ecological momentary assessment in physical activity research. Exerc Sport Sci Rev 2017;45:48–54. 27741022 10.1249/JES.0000000000000092 PMC5161656

[21] Dures E Cramp F Hackett K Primdahl J. Fatigue in inflammatory arthritis. Best Pract Res Clin Rheumatol 2020;34:101526. 32473780 10.1016/j.berh.2020.101526

[22] Dzubur E Ponnada A Nordgren R Yang C-H Intille S Dunton G Hedeker D. MixWILD: a program for examining the effects of variance and slope of time-varying variables in intensive longitudinal data. Behav Res Methods 2020;52:1403–27. 31898295 10.3758/s13428-019-01322-1 PMC7406537

[23] Eyssen IC Steultjens MP Dekker J Terwee CB. A systematic review of instruments assessing participation: challenges in defining participation. Arch Phys Med Rehabil 2011;92:983–97. 21621675 10.1016/j.apmr.2011.01.006

[24] Fawole HO Dell'Isola A Steultjens MP Riskowski JL Chastin SF. Temporal associations between physical activity, mental activity and fatigue dimensions in knee osteoarthritis: an exploratory intensive longitudinal study. Fatigue Biomed Health Behav 2020;8:32–48.

[25] Fawole HO Riskowski JL Dell'Isola A Steultjens MP Nevitt MC Torner JC Lewis CE Felson DT Chastin SFM. Determinants of generalized fatigue in individuals with symptomatic knee osteoarthritis: the MOST Study. Int J Rheum Dis 2020;23:559–68. 31991526 10.1111/1756-185X.13797 PMC7160026

[26] Fertelli TK Tuncay FO. Fatigue in individuals with knee osteoarthritis: its relationship with sleep quality, pain and depression. Pak J Med Sci 2019;35:1040–4. 31372139 10.12669/pjms.35.4.383 PMC6659093

[27] Fillingim RB. Individual differences in pain: understanding the mosaic that makes pain personal. PAIN 2017;158(suppl 1):S11–8. 27902569 10.1097/j.pain.0000000000000775 PMC5350021

[28] Focht BC Ewing V Gauvin L Rejeski WJ. The unique and transient impact of acute exercise on pain perception in older, overweight, or obese adults with knee osteoarthritis. Ann Behav Med 2002;24:201–10. 12173677 10.1207/S15324796ABM2403_05

[29] Fritz H Tarraf W Saleh DJ Cutchin MP. Using a smartphone-based ecological momentary assessment protocol with community dwelling older African Americans. J Gerontol B Psychol Sci Soc Sci 2017;72:876–87. 28057696 10.1093/geronb/gbw166 PMC5927156

[30] Guillemin F Ricatte C Barcenilla-Wong A Schoumacker A Cross M Alleyrat C Buttel T Cembalo M Manseur H Urban H Fautrel B Conaghan PG Hawker G Rutherford C March L Spitz E Hunter DJ. Developing a preliminary definition and domains of flare in knee and hip osteoarthritis (OA): consensus building of the flare-in-OA OMERACT Group. J Rheumatol 2019;46:1188–91. 31092709 10.3899/jrheum.181085

[31] Hamaker EL. Why researchers should think “within-person”: a paradigmatic rationale. Handbook of research methods for studying daily life. New York: The Guilford Press, 2012. pp. 43–61.

[32] Hamilton K White KM Cuddihy T. Using a single-item physical activity measure to describe and validate parents' physical activity patterns. Res Q Exerc Sport 2012;83:340–5. 22808720 10.1080/02701367.2012.10599865

[33] Hedeker D Dunton G. MIXWILD user's guide. Mixed model analysis with intensive longitudinal data, 2018. pp. 75.

[34] Hedeker D Mermelstein R. Modeling variation in intensive longitudinal data, MixWILD: A freeware program for mixed model analysis with intensive longitudinal data, 2020 [in preparation].

[35] Hedeker D Mermelstein RJ Demirtas H. Modeling between‐subject and within‐subject variances in ecological momentary assessment data using mixed‐effects location scale models. Stat Med 2012;31:3328–36. 22419604 10.1002/sim.5338 PMC3655706

[36] Hegarty RS Conner TS Stebbings S Treharne GJ. Feel the fatigue and be active anyway: physical activity on high‐fatigue days protects adults with arthritis from decrements in same‐day positive mood. Arthritis Care Res (Hoboken) 2015;67:1230–6. 25776343 10.1002/acr.22582

[37] Hegarty RS Treharne GJ Stebbings S Graham K Conner TS. Optimising daily diary questionnaires about fatigue, psychological flexibility and well-being: perspectives of people with rheumatic disease. Psychol Health 2019;34:181–99. 30736707 10.1080/08870446.2018.1520232

[38] Helminen E-E Arokoski JP Selander TA Sinikallio SH. Multiple psychological factors predict pain and disability among community-dwelling knee osteoarthritis patients: a five-year prospective study. Clin Rehabil 2020;34:404–15. 31965830 10.1177/0269215519900533

[39] Helminen E-E Sinikallio SH Valjakka AL Väisänen-Rouvali RH Arokoski JP. Determinants of pain and functioning in knee osteoarthritis: a one-year prospective study. Clin Rehabil 2016;30:890–900. 27496698 10.1177/0269215515619660 PMC4976658

[40] Hunt MA Birmingham TB Skarakis-Doyle E Vandervoort AA. Towards a biopsychosocial framework of osteoarthritis of the knee. Disabil Rehabil 2008;30:54–61. 17852218 10.1080/09638280701189960

[41] Hutchings A Calloway M Choy E Hooper M Hunter DJ Jordan JM Zhang Y Baser O Long S Palmer L. The Longitudinal Examination of Arthritis Pain (LEAP) study: relationships between weekly fluctuations in patient-rated joint pain and other health outcomes. J Rheumatol 2007;34:2291–300. 17937461

[42] Iijima H Aoyama T Fukutani N Isho T Yamamoto Y Hiraoka M Miyanobu K Jinnouchi M Kaneda E Kuroki H Matsuda S. Psychological health is associated with knee pain and physical function in patients with knee osteoarthritis: an exploratory cross-sectional study. BMC Psychol 2018;6:19. 29716654 10.1186/s40359-018-0234-3 PMC5930799

[43] Jääskeläinen R. Think-aloud protocol. Handbook translation Stud 2010;1:371–4.

[44] Jaremka LM Andridge RR Fagundes CP Alfano CM Povoski SP Lipari AM Agnese DM Arnold MW Farrar WB Yee LD Carson WE III Bekaii-Saab T Martin EW Jr Schmidt CR Kiecolt-Glaser JK. Pain, depression, and fatigue: loneliness as a longitudinal risk factor. Health Psychol 2014;33:948–57. 23957903 10.1037/a0034012 PMC3992976

[45] Jaremka LM Fagundes CP Glaser R Bennett JM Malarkey WB Kiecolt-Glaser JK. Loneliness predicts pain, depression, and fatigue: understanding the role of immune dysregulation. Psychoneuroendocrinology 2013;38:1310–7. 23273678 10.1016/j.psyneuen.2012.11.016 PMC3633610

[46] Karayannis NV Baumann I Sturgeon JA Melloh M Mackey SC. The impact of social isolation on pain interference: a longitudinal study. Ann Behav Med 2019;53:65–74. 29668841 10.1093/abm/kay017 PMC6301311

[47] Keefe FJ Affleck G France CR Emery CF Waters S Caldwell DS Stainbrook D Hackshaw KV Fox LC Wilson K. Gender differences in pain, coping, and mood in individuals having osteoarthritic knee pain: a within-day analysis. PAIN 2004;110:571–7. 15288397 10.1016/j.pain.2004.03.028

[48] Kittelson AJ George SZ Maluf KS Stevens-Lapsley JE. Future directions in painful knee osteoarthritis: harnessing complexity in a heterogeneous population. Phys Ther 2014;94:422–32. 24179141 10.2522/ptj.20130256 PMC3967122

[49] KU Leuven. m-Path: blended care made easy, 2020. Available at: https://m-path.io/landing/. Accessed January 30, 2021.

[50] Lahtinen O Salmivalli C. The relationship between mindfulness meditation and well-being during 8 weeks of ecological momentary assessment. Mindfulness 2020;11:255–63.

[51] Lee C-H Giuliani F. The role of inflammation in depression and fatigue. Front Immunol 2019;10:1696. 31379879 10.3389/fimmu.2019.01696 PMC6658985

[52] Lee PH Macfarlane DJ Lam TH Stewart SM. Validity of the international physical activity questionnaire short form (IPAQ-SF): a systematic review. Int J Behav Nutr Phys Act 2011;8:115. 22018588 10.1186/1479-5868-8-115 PMC3214824

[53] Lester HF Cullen-Lester KL Walters RW. From nuisance to novel research questions: using multilevel models to predict heterogeneous variances. Organizational Res Methods 2021;24:342–88.

[54] Lewis B Lewis D Cumming G. Frequent measurement of chronic pain: an electronic diary and empirical findings. PAIN 1995;60:341–7. 7596631 10.1016/0304-3959(94)00143-3

[55] Liao Y Skelton K Dunton G Bruening M. A systematic review of methods and procedures used in ecological momentary assessments of diet and physical activity research in youth: an adapted STROBE Checklist for Reporting EMA Studies (CREMAS). J Med Internet Res 2016;18:e151. 27328833 10.2196/jmir.4954 PMC4933800

[56] Littman AJ White E Satia JA Bowen DJ Kristal AR. Reliability and validity of 2 single-item measures of psychosocial stress. Epidemiology 2006;17:398–403. 16641618 10.1097/01.ede.0000219721.89552.51

[57] Louati K Berenbaum F. Fatigue in chronic inflammation—a link to pain pathways. Arthritis Res Ther 2015;17:254. 26435495 10.1186/s13075-015-0784-1 PMC4593220

[58] Lowry V Ouellet P Vendittoli P-A Carlesso LC Wideman TH Desmeules F. Determinants of pain, disability, health-related quality of life and physical performance in patients with knee osteoarthritis awaiting total joint arthroplasty. Disabil Rehabil 2018;40:2734–44. 28728444 10.1080/09638288.2017.1355412

[59] Maas CJM Hox JJ. Sufficient sample sizes for multilevel modeling. Methodology 2005;1:86–92.

[60] Madden VJ Kamerman PR Catley MJ Bellan V Russek LN Camfferman D Lorimer Moseley G. Variability in experimental pain studies: nuisance or opportunity? Br J Anaesth 2021;126:e61–4. 33341221 10.1016/j.bja.2020.11.005 PMC8014940

[61] Maher JP Rebar AL Dunton GF. Ecological momentary assessment is a feasible and valid methodological tool to measure older adults' physical activity and sedentary behavior. Front Psychol 2018;9:1485. 30158891 10.3389/fpsyg.2018.01485 PMC6104625

[62] Marquet O Alberico C Hipp AJ. Pokémon GO and physical activity among college students. A study using Ecological Momentary Assessment. Comput Hum Behav 2018;81:215–22.

[63] Mathew J Adhia DB Smith ML De Ridder D Mani R. Protocol for a pilot randomized sham-controlled clinical trial evaluating the feasibility, safety, and acceptability of infraslow electroencephalography neurofeedback training on experimental and clinical pain outcomes in people with chronic painful knee. NeuroRegulation 2020;7:30–44.

[64] May M Junghaenel DU Ono M Stone AA Schneider S. Ecological momentary assessment methodology in chronic pain research: a systematic review. J Pain 2018;19:699–716. 29371113 10.1016/j.jpain.2018.01.006 PMC6026050

[65] Mehl MR Conner TS. Handbook of research methods for studying daily life. New York: Guilford Press, 2012.

[66] Mestdagh M Verdonck S Piot M Niemeijer K Kilani G Tuerlinckx F Kuppens P Dejonckheere E. m-Path: an easy-to-use and highly tailorable platform for ecological momentary assessment and intervention in behavioral research and clinical practice. Front Digit Health 2023;5:1182175 37920867 10.3389/fdgth.2023.1182175 PMC10619650

[67] Milani SA Marsiske M Cottler LB Chen X Striley CW. Optimal cutoffs for the Montreal Cognitive Assessment vary by race and ethnicity. Alzheimers Dement (Amst) 2018;10:773–81. 30505927 10.1016/j.dadm.2018.09.003 PMC6247398

[68] Montoya AK. Selecting a within- or between-subject design for mediation: validity, causality, and statistical power. Multivariate Behav Res 2022;58:616–36. 35679239 10.1080/00273171.2022.2077287

[69] Murphy SL Kratz AL Williams DA Geisser ME. The association between symptoms, pain coping strategies, and physical activity among people with symptomatic knee and hip osteoarthritis. Front Psychol 2012;3:326. 22969747 10.3389/fpsyg.2012.00326 PMC3432514

[70] Murphy SL Schepens Niemiec S Lyden AK Kratz AL. Pain, fatigue, and physical activity in osteoarthritis: the moderating effects of pain-and fatigue-related activity interference. Arch Phys Med Rehabil 2016;97:S201–9. 27207435 10.1016/j.apmr.2015.05.025

[71] Murphy SL Smith DM. Ecological measurement of fatigue and fatigability in older adults with osteoarthritis. J Gerontol A Biol Sci Med Sci 2010;65:184–9. 19776216 10.1093/gerona/glp137 PMC2904590

[72] Murphy SL Smith DM Clauw DJ Alexander NB. The impact of momentary pain and fatigue on physical activity in women with osteoarthritis. Arthritis Rheum 2008;59:849–56. 18512720 10.1002/art.23710 PMC3046423

[73] Nasreddine ZS Phillips NA Bédirian V Charbonneau S Whitehead V Collin I Cummings JL Chertkow H. The Montreal Cognitive Assessment, MoCA: a brief screening tool for mild cognitive impairment. J Am Geriatr Soc 2005;53:695–9. 15817019 10.1111/j.1532-5415.2005.53221.x

[74] National Clinical Guideline C. National Institute for Health and Clinical Excellence: Guidance. Osteoarthritis: Care and management in adults. London: National Institute for Health and Care Excellence (UK) Copyright © National Clinical Guideline Centre, 2014.

[75] Nicolson PJA Williamson E Morris A Sanchez-Santos MT Bruce J Silman A Lamb SE. Musculoskeletal pain and loneliness, social support and social engagement among older adults: analysis of the Oxford Pain, Activity and Lifestyle cohort. Musculoskeletal Care 2021;19:269–77. 33201582 10.1002/msc.1526 PMC8518502

[76] Overton M Ward S Swain N Falling C Gwynne-Jones D Fillingim R Mani R. Are ecological momentary assessments of pain valid and reliable? A systematic review and meta-analysis. Clin J Pain 2023;39:29–40. 36524770 10.1097/AJP.0000000000001084

[77] Pagé MG Gauvin L Sylvestre M-P Nitulescu R Dyachenko A Choinière M. An ecological momentary assessment study of pain intensity variability: ascertaining extent, predictors, and associations with quality of life, interference and health care utilization among individuals living with chronic low back pain. J Pain 2022;23:1151–66. 35074499 10.1016/j.jpain.2022.01.001

[78] Park H-M Kim H-S Lee Y-J. Knee osteoarthritis and its association with mental health and health-related quality of life: a nationwide cross-sectional study. Geriatr Gerontol Int 2020;20:379–83. 32037727 10.1111/ggi.13879

[79] Parmelee PA Tighe CA Dautovich ND. Sleep disturbance in osteoarthritis: linkages with pain, disability, and depressive symptoms. Arthritis Care Res (Hoboken) 2015;67:358–65. 25283955 10.1002/acr.22459 PMC4342277

[80] Parry E Ogollah R Peat G. Significant pain variability in persons with, or at high risk of, knee osteoarthritis: preliminary investigation based on secondary analysis of cohort data. BMC Musculoskelet Disord 2017;18:80. 28196504 10.1186/s12891-017-1434-3 PMC5310083

[81] Parry E Ogollah R Peat G. ‘Acute flare-ups’ in patients with, or at high risk of, knee osteoarthritis: a daily diary study with case-crossover analysis. Osteoarthritis Cartilage 2019;27:1124–8. 30995523 10.1016/j.joca.2019.04.003

[82] Pascale A Sisler I Smith W Valrie C. Intraindividual pain variability metrics for youth with sickle cell disease: relations to health outcomes. Pediatr Blood Cancer 2023;70:e30194. 36605027 10.1002/pbc.30194 PMC9974742

[83] Petrov ME Goodin BR Cruz-Almeida Y King C Glover TL Bulls HW Herbert M Sibille KT Bartley EJ Fessler BJ Sotolongo A Staud R Redden D Fillingim RB Bradley LA. Disrupted sleep is associated with altered pain processing by sex and ethnicity in knee osteoarthritis. J Pain 2015;16:478–90. 25725172 10.1016/j.jpain.2015.02.004 PMC4424160

[84] Rullier L Atzeni T Husky M Bouisson J Dartigues JF Swendsen J Bergua V. Daily life functioning of community‐dwelling elderly couples: an investigation of the feasibility and validity of Ecological Momentary Assessment. Int J Methods Psychiatr Res 2014;23:208–16. 24375556 10.1002/mpr.1425 PMC6878448

[85] Sabir S Stephanie MJ Chua SK. Factors associated with generalised fatigue among individuals with knee osteoarthritis: a systematic review. Malays J Med Health Sci 2021;17(suppl 3):352–9.

[86] Sasaki E Ota S Chiba D Kimura Y Sasaki S Ando M Yamamoto Y Tsuda E Ishibashi Y. Association between central sensitization and increasing prevalence of nocturnal knee pain in the general population with osteoarthritis from the Iwaki Cohort Study. J Pain Res 2021;14:2449–58. 34413679 10.2147/JPR.S318038 PMC8370489

[87] Schneider S Junghaenel DU Broderick JE Ono M May M Stone AA. II. Indices of pain intensity derived from ecological momentary assessments and their relationships with patient functioning: an individual patient data meta-analysis. J Pain 2021;22:371–85. 33203516 10.1016/j.jpain.2020.10.002 PMC8043976

[88] Schneider S Junghaenel DU Keefe FJ Schwartz JE Stone AA Broderick JE. Individual differences in the day-to-day variability of pain, fatigue, and well-being in patients with rheumatic disease: associations with psychological variables. PAIN 2012;153:813–22. 22349917 10.1016/j.pain.2012.01.001 PMC3307888

[89] Sharma A Kudesia P Shi Q Gandhi R. Anxiety and depression in patients with osteoarthritis: impact and management challenges. Open Access Rheumatol Res Rev 2016;8:103–13. 10.2147/OARRR.S93516 PMC5098683 27843376

[90] Sherman AM. Social relations and depressive symptoms in older adults with knee osteoarthritis. Soc Sci Med 2003;56:247–57. 12473311 10.1016/s0277-9536(02)00023-0

[91] Shiffman S. Designing protocols for ecological momentary assessment. In: Stone AA Shiffman SS Atienza AA Nebeling L, editors. The science of real-time data capture: Self-reports in health research. New York: Oxford University Press, 2007. pp. 27–53.

[92] Shiffman S Stone AA Hufford MR. Ecological momentary assessment. Annu Rev Clin Psychol 2008;4:1–32. 18509902 10.1146/annurev.clinpsy.3.022806.091415

[93] Siviero P Veronese N Smith T Stubbs B Limongi F Zambon S Dennison EM Edwards M Cooper C Timmermans EJ van Schoor NM van der Pas S Schaap LA Denkinger MD Peter R Herbolsheimer F Otero Á Castell MV Pedersen NL Deeg DJH Maggi S, EPOSA Research Group. Association between osteoarthritis and social isolation: data from the EPOSA study. J Am Geriatr Soc 2020;68:87–95. 31529624 10.1111/jgs.16159 PMC6954097

[94] Smith DM Parmelee PA. Within‐day variability of fatigue and pain among African Americans and Non‐Hispanic Whites with osteoarthritis of the knee. Arthritis Care Res (Hoboken) 2016;68:115–22. 26315851 10.1002/acr.22690 PMC4780570

[95] Smith MT Quartana PJ Okonkwo RM Nasir A. Mechanisms by which sleep disturbance contributes to osteoarthritis pain: a conceptual model. Curr Pain Headache Rep 2009;13:447–54. 19889286 10.1007/s11916-009-0073-2

[96] Smith TO Dainty JR Williamson E Martin KR. Association between musculoskeletal pain with social isolation and loneliness: analysis of the English Longitudinal Study of Ageing. Br J Pain 2019;13:82–90. 31019689 10.1177/2049463718802868 PMC6463349

[97] Smyth JM Smyth JM. Ecological momentary assessment research in behavioral medicine. J Happiness Stud 2003;4:35–52.

[98] Stone AA Broderick JE Shiffman SS Schwartz JE. Understanding recall of weekly pain from a momentary assessment perspective: absolute agreement, between-and within-person consistency, and judged change in weekly pain. PAIN 2004;107:61–9. 14715390 10.1016/j.pain.2003.09.020

[99] Stone AA Obbarius A Junghaenel DU Wen CK Schneider S. High-resolution, field approaches for assessing pain: ecological momentary assessment. PAIN 2021;162:4–9. 32833794 10.1097/j.pain.0000000000002049 PMC7737856

[100] Stone AA Shiffman S. Capturing momentary, self-report data: a proposal for reporting guidelines. Ann Behav Med 2002;24:236–43. 12173681 10.1207/S15324796ABM2403_09

[101] Stone AA Shiffman S Schwartz JE Broderick JE Hufford MR. Patient compliance with paper and electronic diaries. Control Clin Trials 2003;24:182–99. 12689739 10.1016/s0197-2456(02)00320-3

[102] Wang X Hunter D Xu J Ding C. Metabolic triggered inflammation in osteoarthritis. Osteoarthritis Cartilage 2015;23:22–30. 25452156 10.1016/j.joca.2014.10.002

[103] Whibley D Braley TJ Kratz AL Murphy SL. Transient effects of sleep on next-day pain and fatigue in older adults with symptomatic osteoarthritis. J Pain 2019;20:1373–82. 31085335 10.1016/j.jpain.2019.04.011

[104] Wilkie R Peat G Thomas E Hooper H Croft PR. The Keele Assessment of Participation: a new instrument to measure participation restriction in population studies. Combined qualitative and quantitative examination of its psychometric properties. Qual Life Res 2005;14:1889–99. 16155776 10.1007/s11136-005-4325-2

[105] Wolf LD Davis MC. Loneliness, daily pain, and perceptions of interpersonal events in adults with fibromyalgia. Health Psychol 2014;33:929–37. 25180546 10.1037/hea0000059 PMC4214136

[106] Yang S-Y Woon EYS Griva K Tan BY. A qualitative study of psychosocial factors in patients with knee osteoarthritis: insights learned from an Asian population. Clin Orthop Relat Res 2023;481:874–84. 36580492 10.1097/CORR.0000000000002526 PMC10097569

[107] Zhaoyang R Martire LM Sliwinski MJ. Morning self-efficacy predicts physical activity throughout the day in knee osteoarthritis. Health Psychol 2017;36:568–76. 28277696 10.1037/hea0000479 PMC5466814

[108] Zheng S Tu L Cicuttini F Zhu Z Han W Antony B Wluka AE Winzenberg T Aitken D Blizzard L Jones G Ding C. Depression in patients with knee osteoarthritis: risk factors and associations with joint symptoms. BMC Musculoskelet Disord 2021;22:40. 33413273 10.1186/s12891-020-03875-1 PMC7791830

